# Home Modifications for Older Adults: The HARP Program

**DOI:** 10.1093/geroni/igaf122.335

**Published:** 2025-12-31

**Authors:** Susan Stark

**Affiliations:** Washington University in St. Louis, St. Louis, Missouri, United States

## Abstract

Occupational therapy (OT) plays an important role in ensuring safe and accessible home environments for aging populations. The Home Hazard Removal Program (HARP) is an OT intervention designed to reduce falls. Initially developed through pilot studies that assessed the feasibility, HARP advanced to clinical trials. Subsequently, the program was implemented within the Long-Term Services and Supports (LTSS) network, demonstrating its scalability and effectiveness in community settings. The current phase of HARP development involves its adaptation for individuals aging with long-term disabilities, addressing the complex needs of a diverse aging population. This series of studies, progressing through the translational continuum, underscores the importance of interdisciplinary collaboration and team science in the development and dissemination of innovative OT interventions. Collaborative efforts among occupational therapists, gerontologists, public health experts, and other stakeholders have been integral to refining HARP’s methodology and achieving measurable improvements in home safety outcomes. These findings provide a valuable roadmap for emerging scholars and professionals seeking to implement evidence-based strategies in gerontology and occupational therapy. This presentation delineates the evolution of HARP through pilot testing, clinical trials, and LTSS network implementation and describes barriers to implementation.

